# Overcoming Historical Barriers: Enhancing Positive Perceptions of Medical Research Among African Americans Through a Conference-Based Workshop

**DOI:** 10.1007/s11606-021-06736-2

**Published:** 2021-06-14

**Authors:** LaPrincess C. Brewer, Maarya Pasha, Pernessa Seele, Sumedha Penheiter, Richard White, Floyd Willis, Monica Albertie, Sarah M. Jenkins, Christopher Pullins

**Affiliations:** 1 Department of Cardiovascular Medicine, Mayo Clinic College of Medicine, Rochester, MN USA; 2 Center for Health Equity and Community Engagement Research, Mayo Clinic, Rochester, MN USA; 3 Department of General Internal Medicine, Mayo Clinic College of Medicine, Rochester, MN USA; 4 The Balm in Gilead, Incorporated, Midlothian, VA USA; 5 Department of Community Internal Medicine, Mayo Clinic, Jacksonville, FL USA; 6 Department of Family Medicine, Mayo Clinic, Jacksonville, FL USA; 7 Center for Health Equity and Community Engagement Research, Mayo Clinic, Jacksonville, FL USA; 8 Department of Health Sciences Research, Mayo Clinic, Rochester, MN USA; 9 Department of Family Medicine, Mayo Clinic Arizona, Scottsdale, AZ USA

**Keywords:** research participation, African Americans, faith-based organizations, health disparities, biomedical research

## Abstract

**Background:**

African Americans (AAs) and other racial/ethnic minority groups continue to be underrepresented in medical research and clinical trials. Failure to create more racially diverse research cohorts can exacerbate existing health disparities among these groups.

**Objective:**

To investigate best practices and strategies for enhancing participation of AAs in medical research among attendees of a preconference Institute at a faith-based public health conference.

**Design:**

Qualitative study using semi-structured interviews.

**Participants:**

A total of 21 out of 29 attendees (90% AA) of the Institute (72% response rate).

**Approach:**

A culturally tailored preconference Institute was held at the 2017 Healthy Churches 2020 National Conference. The Institute was led by AA researchers focused on underrepresentation of AAs in medical research. Semi-structured interviews were conducted 1-year post-Institute (*n*=21) and were audio-recorded, transcribed verbatim, and reviewed using thematic analysis.

**Key Results:**

The majority of attendees reported that they were more likely to participate in medical research after attending the Institute (75%). Salient learning points reported by attendees demonstrated attainment of the Institute objectives. Key themes emerged describing barriers preventing AAs from participating in medical research including fear/lack of trust, lack of information on research projects, and not being approached to participate. Key themes regarding facilitators for participation in medical research by AAs were clear communication of study objectives and research benefits along with trust in researchers.

**Conclusions:**

Attendees’ perceptions of participation in medical research were largely positive following their attendance at a conference-based Institute aimed to address the underrepresentation of AAs in medical research. Our culturally tailored approach to disseminating knowledge of the research process could extend to other national conferences prioritizing AAs and other racial/ethnic minority populations to improve research participation.

**Supplementary Information:**

The online version contains supplementary material available at 10.1007/s11606-021-06736-2.

## INTRODUCTION

African Americans (AAs), among other racial/ethnic minorities, continue to be underrepresented in medical research and clinical trials. This remains the case notwithstanding the 1993 Revitalization Act of the National Institutes of Health (NIH) that mandated all federal grants for clinical research to include women and minorities in research trials.^1, 2^ AAs represent 13% of the US population and only 5% of clinical trial participants.^3, 4^ Despite this underrepresentation, previous studies have shown that AAs have an interest to participate in a variety of medical research studies beyond traditional clinical trials including medical record review, interview, and biobank/genetic studies.^5, 6^ The failure to create a more racially diverse research cohort perpetuates existing health and healthcare disparities which disproportionately affect AAs.

Several historical barriers have led to low participation rates among AAs in clinical trials, including culturally insensitive research models, the lack of affinity with researchers, and the failure to disseminate information on the research process.^7, 8^ Compounding this mistrust in research is the belief in an extractive relationship between researchers and the community, where researchers take data to advance their research aims without communicating findings or translating them for the benefit of the community.^9^

Rivers and colleagues^10^ identified five vital elements to AA’s participation in clinical trials and one largely under-recognized element was the influence of faith on participation. The review showed that those studies acknowledging AA faith beliefs had better participation rates, and furthering that awareness of these beliefs is important to incorporate in recruitment strategies. In the Religious Landscape Study by the Pew Research Center,^11^ 75% of AAs reported religion as vital in their lives, with the majority (83%) regularly attending religious services. The Black Church has been instrumental in unifying the collective voices of the AA community and mobilizing the community behind some of the most influential advocacy and social justice movements of American history.^12^ By contextualizing health within religious tenets, it has synergized healing with faith and enhanced uptake of health initiatives in the community. Health programs made in collaboration with the AA faith community have led to improved health literacy, preventive care, and chronic disease care.^13–20^ Given its centrality in AA life and impact on the health and well-being of the community, the Black church is uniquely positioned to be a valuable partner in enhancing research participation within the AA community.^21–26^ While extant literature has demonstrated the benefits of church-based partnerships for increasing research participation at the individual church level, questions still remain regarding perceptions of medical research across broader AA faith communities.

Our study probed this question by exploring how a national faith-based public health conference with predominantly AA attendees could serve as an avenue to disseminate information on the research process and enhance positive perceptions of medical research. The Healthy Churches 2020 National Conference (Healthy Churches 2020) is a strategic program of The Balm in the Gilead, Incorporated (Inc.), a nonprofit organization founded in 1989 to build and strengthen faith communities and health ministries to deliver programs to improve the health status of people of African descent in the USA and abroad.^27^ The conference is a well-respected national platform with a diversity of attendees representing 17 national denominations and 22 states as well as rural and urban geographic regions across the USA. The conference is designed to facilitate collaboration between public health and healthcare professionals and the AA faith community to disseminate health information, share best practices, and unite in efforts to promote health and wellness. The Balm in Gilead, Inc. is also committed to increasing research participation among AAs through innovative frameworks including community-based workshops at its annual conference. With this in mind, the formation of an Institute on best practices and strategies for recruitment of AAs into medical research was deemed a potentially scalable means to facilitate meaningful discussions on the research process and alter attitudes towards research participation among a wide array of attendees.

The overall study objective was to investigate best practices and strategies for enhancing the participation of AAs in medical research among attendees of a preconference Institute at a faith-based public health conference. The study aimed to determine whether our Institute learning objectives were met by (1) evaluation of attendees’ participation in medical research, (2) assessment of attendees’ message retention, and (3) identification of salient barriers/facilitators to participation in medical research by AAs.

## METHODS

### Development of the Institute

Mayo Clinic–affiliated clinical investigators and community partners from Mayo Clinic enterprise sites [Rochester, MN; Scottsdale, AZ; Jacksonville, FL] initially attended Healthy Churches 2020 in 2015 to increase community engagement and research efforts to address health disparities within the AA community. Since then, there has been a partnership between Mayo Clinic and The Balm in Gilead, Inc. resulting in several successful projects advancing health promotional activities among conference attendees.^18^ In 2017, Mayo Clinic clinical investigators (LB, CP) were invited by The Balm in Gilead, Inc. leadership (PS) to lead an Institute at Health Churches 2020 titled, “Recruiting African-Americans to Participate in Medical Research: Methods, Models and Experiences”, in Hilton Head, South Carolina.^27^ The culturally tailored Institute was led and presented by AA researchers of the same faith background as the audience. The 3-hour Institute was structured as an interactive workshop including succinct lectures, breakout sessions, and a panel discussion (see Appendix A). There were a variety of topics discussed with an overarching focus on best practices in recruitment and retention of AAs in research by highlighting community-engaged research collaborations at Mayo Clinic. Featured presentations were the following: Current state of underrepresentation of African-Americans in medical research, The Researcher Experience: Recruitment strategies to increase enrollment of African-Americans into medical research, and The Participant Experience: Best practices to foster enrollment of African-Americans in medical research. The Institute was culturally tailored in that research studies prioritizing various sectors and organizations of the AA community were highlighted (e.g., faith-based organizations, AA women civic organizations [The Links, Incorporated], rural community). A panel of community partners of Mayo Clinic investigators added an in-depth discussion on the AA participant’s perspective and experience with the research process. The session was designed as a community-based intervention to increase awareness about the importance of research participation among AAs and to promote actual research participation among the attendees.

The Institute learning objectives aimed to empower attendees to:

1. Discuss the current state of underrepresentation of AAs in medical research
2. Understand the importance of diversity in medical research to address health disparities among AAs
3. Identify barriers and facilitators to participation in medical research by AAs
4. Implement innovative, culturally-appropriate recruitment strategies to increase enrollment of AAs into medical research studies
5. Form diverse, interdisciplinary research teams to bolster the participation of AAs in medical research

### Study Design and Participants

The Mayo Clinic Institutional Review Board approved the study protocol as a qualitative study involving semi-structured interviews.^28–30^

All attendees of the Healthy Churches 2020 conference were allowed to select a preconference Institute of interest to attend among an array of topics (e.g., establishing effective church health ministries, health disparities, pastor self-care). There was also a random assignment of attendees by the conference organizers based on available participant slots. All preconference Institutes were promoted by The Balm in Gilead, Inc. through flyers (see Appendix B) but no formal recruitment took place to attend our Institute. There were a total of 29 attendees to our specific Institute on November 14, 2017. Semi-structured interviews were conducted with attendees 1-year following the Institute from November 2018 to December 2018. All 29 attendees of the Institute were invited to participate by email. Twenty-one responded to the invitation and participated in the interviews (72% response rate).

### Procedures

Semi-structured interviews were incorporated to determine if the Institute learning objectives were met. The interview guide was developed with the incorporation of the Institute learning objectives and included both closed and open-ended questions assessing medical research participation among attendees following the Institute and knowledge gained from the Institute regarding the underrepresentation of racial/ethnic minorities in medical research (message retention) as well as their perceived barriers/facilitators to participation in medical research (see Appendix C). Demographic data was also collected (e.g., age, gender, profession). Participants received an initial email from the study team research assistant to coordinate a convenient time for the telephone interview. Research assistants conducted 21 telephone interviews and the average length was approximately 15 min each. Interviews were audio-recorded with the permission of the participants and transcribed verbatim. Each participant received a $25 cash card as an incentive.

## MEASURES

### Participation in Medical Research

A number of interview questions probed participation in medical research after the Institute (see Appendix C). One question stated, “After the 3-hour preconference workshop titled Recruiting African-Americans to Participate in Medical Research: Methods, Models, and Experiences on November 14th, 2017, have you participated in any of the following types of medical research?” The answer choices were as follows: biobank, blood or tissue collection as a healthy volunteer, clinical trial, focus group, genetic testing, survey or questionnaire, or any other type of medical research. The intent of these questions was to determine if participating in the Institute led to actual action by attendees.

Likelihood to participate in medical research after the conference was assessed by the following question: “After attending the 3-hour preconference workshop titled Recruiting African-Americans to Participate in Medical Research: Methods, Models, and Experiences on November 14th, 2017, are you: more likely to participate in medical research, less likely to participate in medical research, or did it have no impact at all on whether you will participate in medical research?” Additionally, the participants were asked, if they wanted to, they could easily participate in a medical research study (scale, strongly disagree to strongly agree). They were also asked: “I know how to locate information to help me participate in medical research” (True, False, Don’t Know). The purpose of the above questions was to determine the intention to act by attendees.

### Message Retention

Message retention was assessed in several ways in the interview through a combination of closed and open-ended questions. One question asked: “To the best of your knowledge, is the following statement true or false: African Americans are underrepresented in medical research?” Another question asked: “To the best of your knowledge, is the following statement true or false: Medical research receiving support from the National Institutes of Health requires the inclusion of racial and ethnic minorities and women?” Attendees were also asked to recall two to three points they learned from the Institute.

### Barriers and Facilitators to Medical Research Participation

Attendees provided two to three barriers and facilitators to research participation among AAs based on knowledge gained from the conference and personal reflections of their experiences with the research process.

### Data Analysis

For qualitative analyses, transcripts of responses to open-ended interview questions were analyzed by content analysis and were grouped into themes and conceptual categories independently by two authors (LB, MP).^31^ Themes were extracted based on methods of content analysis by sorting and categorizing responses into three main categories: (1) participation in medical research, (2) message retention, and (3) barriers and facilitators to participation in medical research. Discrepancies were resolved through the involvement of a third author (CP) until consensus on categories was obtained. Illustrative quotes by theme were identified through the coding process. Responses to the closed-ended interview questions (quantitative) were summarized with frequencies and percentages. All statistical analyses (quantitative data) were performed using SAS version 9.4 (SAS Institute Inc., Cary, NC).

## RESULTS

### Participant Characteristics

Table 1 depicts sociodemographics of the 21 interview participants. The mean age was 56 years (range 34–72). Most were AA (90%) and women (71%). Greater than half (52%) had a graduate or professional degree (Master’s/Ph.D./Doctorate). Forty-eight percent reported they were representing a religious or faith-based organization and approximately one third were clergy or health ministry leaders (29%).

**Table 1:** Participant characteristics

	N=21
Age, years	
Mean	56
Range	34-72
Sex	
Women	15 (71%)
Men	6 (29%)
Race	
African-American	19 (90%)
White	1 (5%)
Declined to answer	1 (5)
Marital Status	
Married	14 (67%)
Separated/Divorced	2 (10%)
Single/Never married	5 (24%)
Education	
Graduate or professional degree	11 (52%)
4-year college degree	5 (24%)
Some college	4 (19%)
High school diploma	1 (5%)
Type of Organization Representing	
Religious	10 (48%)
Other	5 (24%)
Academic	4 (19%)
Health care	2 (9%)
Profession^a^	
Healthcare professional	6 (29%)
Clergy	5 (24%)
Retired	5 (24%)
Homemaker	1 (5%)
Health ministry leader	1 (5%)
Other	5 (5%)

^a^Note, selections are not mutually exclusive

### Participation in Medical Research

Thirty-eight percent (8/21) of respondents said they had participated in at least one type of medical research after attending the Institute (Table 2). Most often, that was a focus group (87.5%, 7/8 respondents) and/or a survey or questionnaire (37.5%, 3/8 respondents). The projects were on an array of topics including heart health, opioids, infant mortality, and Alzheimer’s disease. After attending the Institute, 75% (15/20) stated they were more likely to participate in medical research (Table 3). Fifteen of the 21 respondents (71%) agreed or strongly agreed with the statement, “If I wanted to, I could easily participate in a medical research study.”

**Table 2:** Post-Institute attendee participation in medical research by study type (N=8)^a^

Study type	N (%)
Biobank	1/8 (12.5%)
Blood or tissue collection as a healthy volunteer	1/8 (12.5%)
Clinical trial	1/8 (12.5%)
Focus group	7/8 (87.5%)
Genetic testing	2/8 (25.0%)
Survey or questionnaire	3/8 (37.5%)

^a^Note, includes attendees reporting participation in at least one type of medical research study after attending the Institute. Selections are not mutually exclusive

**Table 3:** Post-Institute participation in medical research (N=20)^a^

Interview question	N (%)
More likely to participate in medical research	15/20 (75%)
If I wanted to, I could easily participate in a medical research study (Agree/Strongly Agree)	15/20 (71%)
I know how to locate information to help me participate in medical research (True)	12/20 (57%)

^a^One attendee did not respond to the question

### Message Retention

#### True/False and Multiple-Choice Questions

After attending the workshop, 90% (19/21) of respondents correctly answered the statement, “AAs are underrepresented in medical research,” 90% (19/21) correctly answered “Medical research receiving support from the NIH requires the inclusion of racial and ethnic minorities and women,” and 62% (13/21) correctly answered the question that “5% of research participants in the United States are AA.”

#### Salient Themes Around Message Retention

Attendee responses collectively echoed the importance of becoming a more representative body in medical research, and the implications of the current state of low representation in preventing evidence-based advancements in treatments for specific medical conditions for not only themselves but also for future generations (see Appendix D). Responses also reflected an understanding of the overall impact medical research has on improving health care for their community. The importance of community engagement throughout the research process was emphasized, from initiation to completion, including dissemination of findings. One participant conveyed how he used this information to raise awareness by sharing with his congregation:

The one thing I did learn is that there hadn’t been a great deal of African American participants in clinical trials and research, and I brought that information back to our congregation. [Male, age 72]

Another participant shared the impact the Institute had on changing her mindset about participating in research:

I know I’ve gotten letters being offered to participate, and I just kind of threw them away. But since that workshop, I’m becoming more aware or seeing what’s out there and actually trying to get involved in some of the clinical research. [Female, age 60]

In addition, many attendees recalled key techniques for recruitment of AAs into clinical trials that were mentioned during the Institute. Many highlighted using a community-based approach and the importance of tailoring efforts to particular communities or churches. These techniques resonated with attendees and were felt to be tangible:

And the ways I thought which were really creative in regards to being able to recruit individuals…tailoring the recruitment for that research, having a cultural lens approach in regards to recruiting individuals. [Female, age 48]

Several attendees mentioned the effectiveness of community-based efforts in the research process, indicating the support and resources they believed came from their own communities. They also underscored the importance of building partnerships with trusted community organizations.

I do remember the take-home message of don’t think that you have to provide all of the resources yourself, but try to connect with partners in the community to provide the services. Don’t try to reinvent the wheel. [Female, age 45]

### Barriers and Facilitators to Medical Research Participation

#### Salient Themes Around Barriers

Themes that arose around barriers to participation revolved around fear, lack of trust, lack of knowledge, and historical trauma (see Appendix D). Many respondents stressed the mistrust AA communities have in the medical system as a barrier, which has stemmed largely from historical trauma such as the Tuskegee Syphilis Study. Another theme emerged around AAs not being made aware of available medical research studies or being approached to participate. Participants also mentioned not being equipped with information to make informed decisions on participation.

And then we’re not approached. I’m 72 years old, and I’ve never heard the word ‘clinical trial’ come out of a single doctor’s mouth. Ever. Ever. [Female, age 72]

Furthermore, respondents mentioned researchers often do not meet people where they are in the community and suggested going beyond flyer distribution to increase personal contact with potential participants during the recruitment phase. They emphasized that researchers must clearly define the importance of participation using compelling reasons for why participation matters and relates to participants' health.

#### Salient Themes Around Facilitators

A recurring theme was the importance of trust-building based on transparency in research and using trusted community liaisons (see Appendix D). Another critical factor was to have well-informed researchers, preferably from the AA community. Church endorsement or approval of the clinical trial or particular medical research study was a key facilitator. Furthermore, respondents stressed that researchers must ensure that participants are given avenues to voice study concerns. They also suggested having prior participants give testimonials about their experiences with the clinical trial to recruit new participants. “Closing the loop” on research by bringing the findings back to the community was mentioned as a vital component to keep participants informed beyond the data collection or intervention phases. Participants underscored the importance of researchers clearly communicating benefits to participation, and how this research would affect their communities.

I think many times [it] is your approach in regards to actually educating people about the importance of participating in research studies, how it benefits our society, but also how African-Americans can really suffer from a lot of comorbid diseases, how that’s actually going to impact them and the community as a whole. [Female, age 48]

Participants collectively called for transparency and wanted to be more informed on the entire research process and study findings.

I think one thing is educating people. Being transparent when doing research because a lot of the times people go into communities, they do research, and they take the valuable research. They don’t give anything back; they don’t tell them what the end results were. So being transparent, informing people, truly informing them from the beginning to the end of the process. [Female, age 34]

Trust was a common theme woven throughout several responses, emphasizing the importance of having AA researchers to help participants better identify with those leading studies.

Having researchers that look like African-Americans give their knowledge and how beneficial it will be. I think that would give African-Americans more trust into participating in the research. [Female, age 60]

## DISCUSSION

Our study findings suggest that a culturally tailored Institute on research best practices and recruitment strategies at a national, faith-based public health conference positively influenced views of medical research participation among AAs. One year following the Institute, there was favorable message retention among the attendees of core topics on the research process discussed during the Institute. Furthermore, the attendees were eager to share key learning points with their local health ministries and communities. The majority of the attendees reported a greater likelihood to participate in medical research following the Institute. The distinct placement of the Institute within the context of Healthy Churches 2020 provided an avenue to engage AA faith communities in a setting where health promotion sets the tone for research education in a trusted environment. Our study findings suggest that influencing thought leaders of AA faith communities at a national conference could be a useful strategy to propagate change in attitudes towards medical research at a grassroots level.

Similar to prior investigations, our study highlights the importance of AA researchers leading research efforts through culturally tailored approaches.^26^ Whitt-Glover and colleagues^25^ demonstrated that the most successful recruitment efforts in AA faith communities are led by those of the same faith-based community, those with a history of working in the community in various roles, and those who were trusted community leaders. The research faculty of our Institute are all AA and reputable leaders in several community faith-based platforms, which likely created an environment of trust and facilitated learning by the audience. Furthermore, all speakers shared a similar faith background with the attendees, which fostered a collective sense of values. Based on previous community-based research, cultural congruence is an important factor for successful research partnerships.^7^ Similarly, a recent study discussed the concerns of AA participants and their skepticism of certain researchers given historical unethical research practices.^32^ The authors further highlighted the importance of a shared background and understanding between researchers and participants as was shown in our study.

Our study obtained valuable insights into barriers and facilitators to research participation among AAs. Lack of information and knowledge of the research process was mentioned by many respondents as a barrier to participation consistent with findings from other qualitative studies, strengthening the original intent and objectives of our study.^33^ The attendees reinforced the importance of researchers explicitly stating benefits of participation to the broader community and proactively sharing study findings as facilitators to research participation. Slade and colleagues^21^ demonstrated the effectiveness of these techniques by their dissemination of study findings to church congregation members through purposeful data-sharing sessions. Therefore, the academic research team was able to maintain more sustainable relationships with churches, which facilitated later recruitment into studies.

## Strengths and Limitations

Our study has several strengths. To our knowledge, no studies have demonstrated the success of a research Institute held at a national conference in disseminating medical research information among a larger community of AAs within a diverse network of clergy and health ministry leaders. Our multifaceted, qualitative approach allowed us to gain rich perspectives from a group that has been historically underrepresented in research. Furthermore, we demonstrated a potentially scalable strategy to present information on the research process in a culturally relevant manner. The attendees of our Institute reported that they dispersed what they learned from the Institute to their congregations and communities, demonstrating that our efforts can create a channel for increasing research participation among AAs as well as other racial/ethnic minority groups. Our purposeful yet respectful engagement process with a national faith-based organization to reach the AA community can serve as a model for others to follow to enhance all stages of research, including study design, recruitment, and translation.^34–37^

This study also has limitations that are worth mentioning. We recognize that our sample size was small. However, since our study was exploratory and qualitative in nature, the sample size did suffice in gathering valuable insights on the perceptions towards medical research among AAs. Another limitation is that a convenience sample was used, which may also limit generalizability to the entire AA faith community and to all AAs. Nevertheless, our study sample was comparable to and representative of the Healthy Churches 2020 attendees as a whole.^27^ An additional limitation is that the attendees were potentially already more likely to participate in medical research prior to attending the Institute since they were attending an overarching conference centered on health promotion. Also, we did not conduct a pretest evaluation inclusive of quantitative methods to clarify attitudes about the research process among the attendees prior to the Institute. Thus, causal inferences and impact evaluation could not be determined. Furthermore, approximately half of the attendees (11/21) reported participation in medical research prior to the Institute. Of these attendees, one third (7/21) reported continued participation in research after the Institute. Having previously participated in research could have influenced their likelihood and willingness to participate in future research. Nevertheless, our results strengthen the need for similar workshops to connect AAs to opportunities to participate in medical research. We also did not specifically probe the rationale or reservations from those reporting that they were less likely to participate in research after the Institute. Assessment of this reasoning could be useful information to incorporate into future workshops. We acknowledge this is a self-selected group of individuals, but our study contributes meaningfully to understanding perceptions AA faith communities have towards research.

Our evaluation of the Institute is crucial for future conferences as it can inform quality improvement by the Institute co-chairs and featured speakers to implement similar but enhanced research sessions at other national conferences, including those with large audiences of AAs beyond the faith community as well as other racial/ethnic minority groups. Thus, our findings and the implications of ongoing collaborative efforts are relevant to these diverse populations.

## Conclusion

We present a novel way to enhance positive perceptions of research among AAs in a culturally sensitive manner that underscores the importance of trust-building and community-based participation. The attendees’ perceptions of participation in medical research were largely positive following their attendance at a conference-based Institute aimed to address the underrepresentation of AAs in medical research. The findings of our study suggest that conferences can serve as national platforms to inform and engage AAs and other racial/ethnic minorities on the importance of research participation. These efforts may help advance equitable research towards eliminating health and healthcare disparities.

## Supplementary Information

ESM 1 (PDF 429 kb)

## **Abbreviations**

AA: African American
Healthy Churches 2020: Healthy Churches 2020 National Conference
Inc.: Incorporated
NIH: National Institutes of Health
