# Supplementary material for: Overcoming Historical Barriers: Enhancing Positive Perceptions of Medical Research Among African Americans Through a Conference-Based Workshop
Source: J Gen Intern Med. 2021 Jun 14;36(9):2547–54. doi: 10.1007/s11606-021-06736-2 (PMC8390631; doi:10.1007/s11606-021-06736-2)
Supplement: Supplementary file 1 — (PDF 429 kb) [file 11606_2021_6736_MOESM1_ESM.pdf]

**Appendix A**. Healthy Churches 2020 National Conference pre-conference Institute Agenda

MAYO CLINIC

**Mayo Clinic Office of Health Disparities Research presents:**
*Recruiting African Americans to Participate in Medical Research:*
*Methods, Models and Experiences*
Balm in Gilead, Inc., Healthy Churches 2020 Conference, Hilton Head, SC
Tuesday, November 14, 2017, 2-5pm

**INSTITUTE AGENDA (updated 9/19/2017)**

**2:00-2:15 pm WELCOME AND INTRODUCTION**
*Current state of underrepresentation of African-Americans in medical research*
**LaPrincess Brewer, MD, MPH, Mayo Clinic Rochester**
**Christopher Pullins, MD, Mayo Clinic Arizona**

**2:15-3:15 pm SESSION 1: Part 1**
*The Researcher Experience: Recruitment strategies to increase enrollment of African-Americans into medical research*
**Monica Albertie, Mayo Clinic Florida**
**Richard White, MD, Mayo Clinic Florida**
**Floyd Willis, MD, Mayo Clinic Florida**

**3:15-3:25 pm SESSION 1: Q & A**

**3:25-3:40 pm Break** *(15 minutes)*

**3:40-4:10 pm SESSION 1: Part 2**
*The Researcher Experience: Recruitment strategies to increase enrollment of African-Americans into medical research*
**LaPrincess Brewer, MD, MPH, Mayo Clinic Rochester**
**Christopher Pullins, MD, Mayo Clinic Arizona**

**4:10-4:20 pm SESSION 2: Q & A**

**4:20-4:50 pm SESSION 3: PANEL DISCUSSION**
*The Participant Experience: Best practices to foster enrollment of African-Americans in medical research*
**Mayo Clinic Rochester (Jacqueline Johnson, MSW; Clarence Jones, MEd)**
**Mayo Clinic Florida (Bishop Rudolph McKissick, Sr.)**
**Mayo Clinic Arizona (Angela M. Allen, PhD)**

**4:50–5pm CLOSING REMARKS**
**LaPrincess Brewer, MD, MPH, Mayo Clinic Rochester**
**Christopher Pullins, MD, Mayo Clinic Arizona**

**Appendix B.** Healthy Churches 2020 National Conference pre-conference Institute flyer

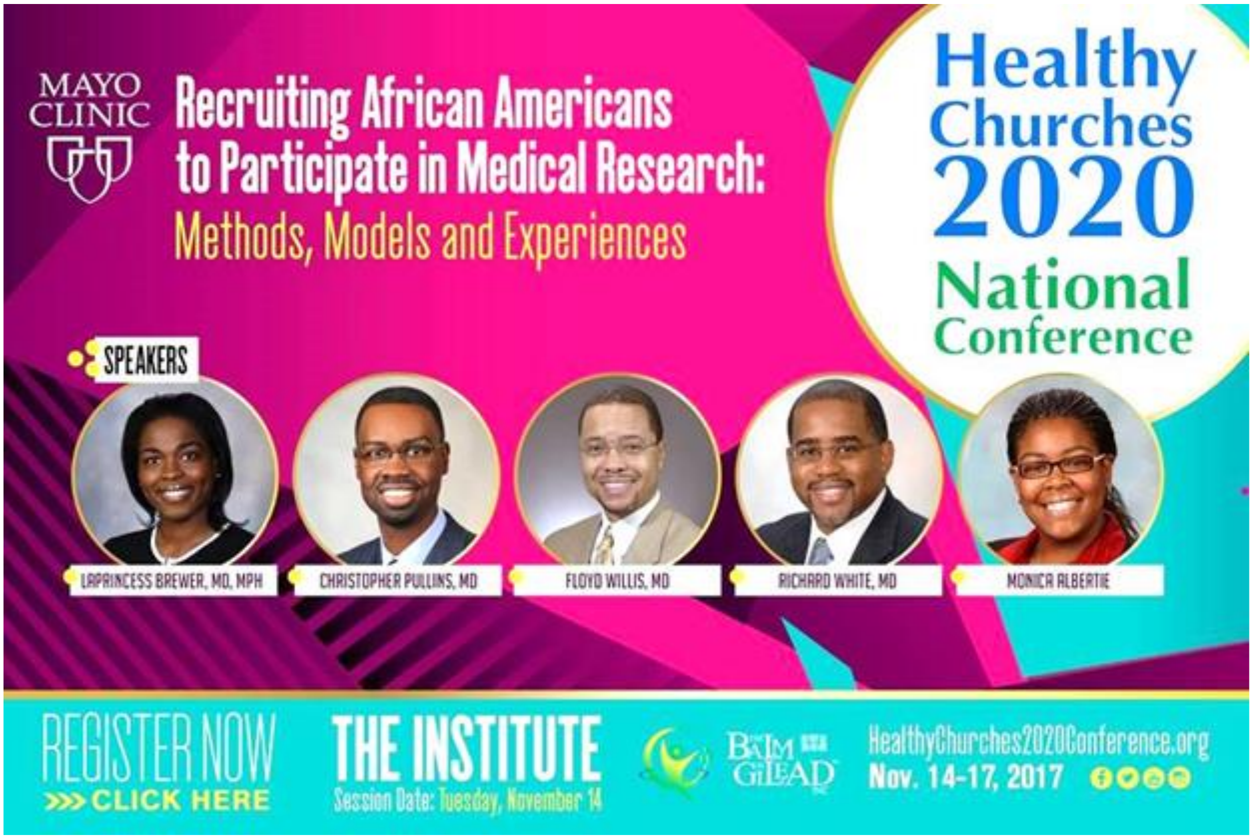

**Appendix C**. Semi-structured interview guide

**JOB M-221** **Mayo Clinic HC2020 Follow-up Research**

**ID#:_____________**

**In-Depth Interview Discussion Guide**

**November, 2018** **INT:_____________**

**ASK TO SPEAK WITH THE CONTACT LISTED. IF UNAVAILABLE, SCHEDULE CALLBACK.**

**Contact Name:** ______________________________________________________________

**Interview Date:** _______**Interview Start Time:** ________ **Interview End Time:** ________

**INTRODUCTION:** Good morning/afternoon. This is [insert interviewer's name]calling from Mayo Clinic.

**READ IF ALREADY AGREED TO PARTICIPATE:**
Recently, you responded to an email from [insert principal investigator's name], principal investigator for a research project titled: Recruiting African-Americans to Participate in Medical Research: Methods, Models, and Experiences. As a participant in the Healthy Churches 2020 National Conference Pre-conference Institute in November of 2017, she invited you to participate in this research project and you replied back indicating your interest in participating. Is now a good time to conduct the interview or would you like to schedule it for another time?

**READ IF HAS NOT ALREADY AGREED TO PARTICIPATE:**
Recently, you should have received an email from [insert principal investigator's name], principal investigator for a research project titled: Recruiting African-Americans to Participate in Medical Research: Methods, Models, and Experiences. As a participant in the Healthy Churches 2020 National Conference Pre-conference Institute in November of 2017, she invited you to participate in this research project. Is now a good time to conduct the interview or would you like to schedule it for another time?

**INTRODUCTION, CONTINUED:** The Mayo Clinic Office of Health Disparities Research is following up with attendees of the Mayo Clinic-led 3-hour pre-conference workshop titled Recruiting African-Americans to Participate in Medical Research: Methods, Models, and Experiences Co-chaired by [insert co-chairs'names] held on November $14^{th}$, 2017.

Just to check, did you attend the 3-hour pre-conference workshop titled Recruiting African-Americans to Participate in Medical Research: Methods, Models, and Experiences Co-chaired by [insert co-chairs' names] on November $14^{th}$, 2017?
**(INTERVIEWER: CHECK ONE RESPONSE.)**

☐ Yes **--> CONTINUE.** ☐ No **--> THANK AND END INTERVIEW.**

We thank you for your attendance of our session and would like to ask a few questions about your experience during and after it. Our goal is to use this information to improve future sessions. This research study is led by physicians and researchers, [insert names] of the Mayo Clinic. The interview

is entirely voluntary and will be recorded to ensure an accurate summary report is developed of what was said, not of who said what. All answers given are entirely confidential; only the study team will review this information. You will receive a $25 gift card for your time, either in person if you are attending this year's Healthy Churches 2020 National Conference or a check by mail if not attending. If you agree to participate, we will move forward with the interview, which will take approximately 15 minutes of your time.

Are you willing to continue with this interview? **(INTERVIEWER: CHECK ONE RESPONSE.)**

☐ Yes **--> CONTINUE.** ☐ No **--> THANK AND END INTERVIEW.**

**INTERVIEWER: START RECORDING!**

1. Prior to attending the 3-hour pre-conference workshop titled Recruiting African-Americans to Participate in Medical Research: Methods, Models, and Experiences Co-chaired by [insert co-chairs' names] on November 14th, 2017, had you ever participated in any of the following types of medical research?
   **(INTERVIEWER: READ LIST. CHECK 'YES' RESPONSES.)**

   ☐ Biobank
   ☐ Blood or tissue collection as a healthy volunteer
   ☐ Clinical trial
   ☐ Focus group
   ☐ Genetic testing
   ☐ Survey or questionnaire
   ☐ Any other type of medical research?
   (SPECIFY:________________________________________)
   ☐ **INTERVIEWER: DO NOT READ BUT VERIFY IF NONE CHECKED ABOVE**—So, none of these?

2. Prior to attending the 3-hour pre-conference workshop titled Recruiting African-Americans to Participate in Medical Research: Methods, Models, and Experiences Co-chaired by [insert co-chairs' names] on November 14th, 2017, had you ever initiated or been a part of a research team for any projects that included African-American research participants? **(INTERVIEWER: CHECK ONE RESPONSE.)**

   ☐ Yes ☐ No ☐ Don't Know

3. After attending the 3-hour pre-conference workshop titled Recruiting African-Americans to Participate in Medical Research: Methods, Models, and Experiences Co-chaired by [insert co-chairs' names] on November 14th, 2017, have you participated in any of the following types of medical research?
   **(INTERVIEWER: READ LIST. CHECK 'YES' RESPONSES.)**

   ☐ Biobank
   ☐ Blood or tissue collection as a healthy volunteer
   ☐ Clinical trial
   ☐ Focus group
   ☐ Genetic testing
   ☐ Survey or questionnaire

☐ Any other type of medical research?
(SPECIFY:_____________________________________)
☐ **INTERVIEWER: DO NOT READ BUT VERIFY IF NONE CHECKED ABOVE**—So, none of these?

4. After attending the 3-hour pre-conference workshop titled Recruiting African-Americans to Participate in Medical Research: Methods, Models, and Experiences Co-chaired by [insert co-chairs' names] on November $14^{th}$, 2017, have you initiated or been a part of a research team for any projects that included African-American research participants? **(INTERVIEWER: CHECK ONE RESPONSE.)**

☐ Yes ☐ No ☐ Don't Know

**INTERVIEWER: ONLY ASK IF 'YES' IN Q4**

5. Will you please describe the research project(s) you initiated or were a part of that included African-American research participants?

**(INTERVIEWER: MAKE SURE THE RESPONSE ANSWERS THE QUESTION. IF IT DOES NOT, RE-ASK. BE SURE TO PROBE FOR A COMPLETE RESPONSE AND THEN CLARIFY FOR UNDERSTANDING.)**

___________________________________________________________________________

___________________________________________________________________________

___________________________________________________________________________

**INTERVIEWER: ONLY ASK IF 'NO' IN Q4**

6. Why haven't you initiated or been a part of a research team for any projects that included African-American research participants in the past year?

**(INTERVIEWER: MAKE SURE THE RESPONSE ANSWERS THE QUESTION. IF IT DOES NOT, RE-ASK. BE SURE TO PROBE FOR A COMPLETE RESPONSE AND THEN CLARIFY FOR UNDERSTANDING.)**

___________________________________________________________________________

___________________________________________________________________________

___________________________________________________________________________

7. Please tell me one or two things you learned from attending the 'Recruiting African-Americans to Participate in Medical Research: Methods, Models, and Experiences' workshop.

**(INTERVIEWER: MAKE SURE THE RESPONSE ANSWERS THE QUESTION. IF IT DOES NOT, RE-ASK. BE SURE TO PROBE FOR A COMPLETE RESPONSE AND THEN CLARIFY FOR UNDERSTANDING.)**

___________________________________________________________________________

___________________________________________________________________________

___________________________________________________________________________

8. To the best of your knowledge, is the following statement true or false: *African-Americans are underrepresented in medical research?*
**(INTERVIEWER: CHECK ONE RESPONSE.)**

☐ True ☐ False ☐ Don't Know

9. To the best of your knowledge, is the following statement true or false: *Medical research receiving support from the National Institutes of Health requires the inclusion of racial and ethnic minorities and women?*
**(INTERVIEWER: CHECK ONE RESPONSE.)**

☐ True ☐ False ☐ Don't Know

10. To the best of your knowledge, what percentage of medical research participants in the United States are African American?
**(INTERVIEWER: CHECK ONE RESPONSE.)**

☐ 5% ☐ 10% ☐ 15% ☐ 20%

11. What are two or three barriers (things getting in the way or preventing) that prevent African Americans from participating in medical research?

**(INTERVIEWER: MAKE SURE THE RESPONSE ANSWERS THE QUESTION. IF IT DOES NOT, RE-ASK. BE SURE TO PROBE FOR A C/OMPLETE RESPONSE AND THEN CLARIFY FOR UNDERSTANDING.)**

______________________________________________________________________

______________________________________________________________________

______________________________________________________________________

12. What are two or three facilitators (things making it easier or helping) that help African Americans participate in medical research?

**(INTERVIEWER: MAKE SURE THE RESPONSE ANSWERS THE QUESTION. IF IT DOES NOT, RE-ASK. BE SURE TO PROBE FOR A COMPLETE RESPONSE AND THEN CLARIFY FOR UNDERSTANDING.)**

______________________________________________________________________

______________________________________________________________________

______________________________________________________________________

13. Do you agree or disagree with the following statement: *If I wanted to, I could easily participate in a medical research study?*
**(INTERVIEWER: AFTER INITIAL RESPONSE, ASK WHETHER STRONGLY OR NOT, THEN CHECK ONE RESPONSE.)**

☐ Strongly agree
☐ Agree
☐ Disagree
☐ Strongly disagree

☐ **DO NOT READ:** Don't Know

14. I know how to locate information to help me participate in medical research.
**(INTERVIEWER: CHECK ONE RESPONSE.)**

☐ True ☐ False ☐ Don't Know

15. After attending the 3-hour pre-conference workshop titled Recruiting African-Americans to Participate in Medical Research: Methods, Models, and Experiences Co-chaired by [insert co-chairs' names] on November 14th, 2017, are you: **(INTERVIEWER: READ LIST. CHECK ONE RESPONSE.)**

☐ More likely to participate in medical research
☐ Less likely to participate in medical research, or
☐ Did it have no impact at all on whether or not you will participate in medical research
☐ **DO NOT READ:** Don't Know

16. How likely is it that you would recommend the workshop titled Recruiting African-Americans to Participate in Medical Research: Methods, Models, and Experiences to a friend or colleague? Please use a zero to ten scale where 0 means not at all likely and 10 means extremely likely.
**(INTERVIEWER: CIRCLE ONE NUMBER.)**

NOT AT ALL
EXTREMELY DK/REF
0 1 2 3 4 5 6 7 8 9 10
99

The last few questions are demographic in nature and will allow the research team to accurately describe the group of survey participants and better analyze the data.

17. What is your current profession? **(INTERVIEWER: READ LIST. CHECK ALL RESPONSES THAT APPLY.)**

☐ Clergy
☐ Health ministry leader
☐ Health care professional
☐ Homemaker
☐ Retired
☐ Other: please specify ________________________________

18. What type of organization did you represent when attending the Healthy Churches 2020 National Conference Pre-conference Institute in November of 2017? **(INTERVIEWER: READ LIST. CHECK ALL RESPONSES THAT APPLY.)**

☐ Academic institution
☐ Health care provider
☐ Religious or faith-based organization

☐ Other: please specify ______________________________

19. What is your current age? **(INTERVIEWER: RECORD NUMBER.)** _____ year(s)

20. What gender do you identify yourself as?
**(INTERVIEWER: READ LIST. CHECK <u>ONE</u> RESPONSE.)**

☐ Female ☐ Male ☐ Other

21. Would you describe your ethnic origin as:
**(INTERVIEWER: READ LIST. CHECK <u>ONE</u> RESPONSE.)**

☐ Hispanic or Latino ☐ Not Hispanic or Latino

22. Which of the following describe your race?
**(INTERVIEWER: READ LIST. CHECK <u>ALL</u> RESPONSES THAT APPLY.)**

☐ African-American
☐ American Indian/American Native
☐ Asian-American
☐ Native Hawaiian/Pacific Islander
☐ White
☐ Other: please specify ______________________________

23. Which of the following best describes your current marital status:
**(INTERVIEWER: READ LIST. CHECK <u>ONE</u> RESPONSE.)**

☐ Never married ☐ Widowed ☐ Living with significant other
☐ Separated/Divorced ☐ Married ☐ Other: please specify____

24. What is the highest level of formal education you have completed?
**(INTERVIEWER: READ LIST. CHECK <u>ONE</u> RESPONSE.)**

☐ Less than 8th grade
☐ Completed 8th grade
☐ Some high school
☐ Graduated high school
☐ GED or ABE certificate
☐ Some college, technical, vocational school (AA, LPN, etc.) but not a 4-year degree
☐ Graduated with a 4-year college degree (BS, BA, etc.)
☐ Graduate or professional degree: please specify ________________________
☐ Other: please specify_________________________

25. Are you planning to attend this year's Healthy Churches 2020 National Conference being held in Point Clear, Alabama November 13th through the 16th? **(INTERVIEWER: CHECK <u>ONE</u> RESPONSE.)**

☐ Yes **-->** Great! **(INTERVIEWER: EXPLAIN TO RESPONDENT HOW TO CLAIM THEIR GIFT CARD, THEN SKIP TO CLOSE.)**

☐ No **-->** Can I please get your mailing address so I can send you a check?
**(INTERVIEWER: RECORD ADDRESS BELOW.)**

**STREET ADDRESS:**___________________________________________

**CITY:**_____________________________________________________

**STATE AND ZIP CODE:**_______________________________________

**CLOSE: Those are all the questions I have for you today. On behalf of the investigators of this research:** [insert co-chairs' names], **thank you very much for your time and participation!**

**INTERVIEWER: STOP RECORDING!**

| **Interview End Time:** ________ |
| --- |

**Definitions:**

Biobank- A place that stores biological samples for use in research.

Blood or tissue collection as a healthy volunteer- Blood or tissue collected for research purposes.

Clinical trial- Researchers learn if a new test or treatment works and is safe, examples (new drugs, new surgical procedures or devices, or new ways to use existing treatments).

Focus group- Small number of people brought together with a moderator to focus on a specific topic. Focus groups also known as round table discussions or listening sessions aim at a discussion instead of on individual responses to formal questions.

Genetic testing- Involves looking for changes in DNA, genes, or proteins. The results of a genetic test can confirm or rule out a suspected genetic or hereditary condition or help determine a person's chance of developing or passing on a genetic disorder.

Survey or questionnaire- An instrument used to collect a set of answers from participants.

**Appendix D.** Salient themes and selected attendee commentary on Institute message retention and barriers/facilitators to participation in medical research

| Themes | Illustrative quotes |
|---|---|
| **Message Retention** | |
| **Underrepresentation of African-Americans in medical research** | "I must say I did feel like I learned a lot overall from the workshop. This was at the time - and still is - a rather new area for me in terms of work, so I did feel very well-informed when I left the workshop just about overall roadblocks or barriers to clinical trial participation, particularly in the African-American community." [Female, age 35] |
| **Recruitment strategies** | "Well I think one of the things that has always been important has really been the need to have African-Americans to participate in research. The other thing, too, is learning some of the key techniques for that, and then to see how other people have used it, you know, in terms of their own experiences working with various demographics and in various places. Because, you know, the way that you recruit or work with folks in one community doesn't actually work in another community, so it's just kind of good to hear the differences in other communities." [Male, age 66] |
| **Community engagement** | "What I remember is talking a lot about building community through relationships, and I think there was even a mention of an advisory board. Um, and so there was a lot of talk and interest about how to engage the community in those types of ways and keeping them informed throughout the process. So from what I can recall, I believe that was the premise of the discussion." [Female, age 35] |
| **Key Barriers** | |
| **Lack of information/not being approached** | "Well, knowledge is power, and I think whenever someone is asked to be selected, you're taking the time to speak with me one-on-one and I think that's very important that there's more exposure about, you know, about how the information is going to be used to put people at ease so they can understand exactly |

| | |
|---|---|
| | what's going to happen with that information and how it will impact the African-American community." [Female, age 66] |
| **Fear/lack of trust** | "Yeah, I think first of all we're not invited so we don't know. And then those who do know, they're fearful. Fear is a tremendous factor and I don't think we understand. If we know, we may understand that the outcomes could be positive to the next generation of people. So we don't know, there's fear, and we don't understand what the outcomes could be of participating in a study that will make a difference in somebody's life after us. You know, we have to think about the next generation, you know, because they're going to get sick, they're going to have the cancers, they're going to have the illnesses and you can't say that African-American women at a certain group do well on this study, on these drugs when they've never been studied." [Female, age 72] |
| **Historical trauma** | "…it's historical facts, you know, like I said, The Tuskegee experiment.  There was an experiment in Ohio where pregnant black women, where they would sterilize them without their knowledge once they gave birth" [(Female, age >65)] |
| **Key Facilitators** | |
| **Understanding the benefits to research** | "I think many times is your approach in regards to actually educating people about the importance of participating in research studies, how it benefits our society. But also how many times because African-Americans can really suffer from a lot of comorbid diseases, how that's actually going to impact them and impact the community as a whole. So those things I think are very critical. And also being able to… So, educating people in regards to the importance of participating in research is very critical, and then also making sure that you're able to reach those, actually reach the people who you are trying to recruit, too. So making the additional effort of going into those communities. And then also making sure that even after you get the information, that you will go back and share the results of the individual's participation in the research is really critical, too." [Sex, age not given] |

| | |
|---|---|
| **Information/being informed** | "I think being fully or adequately informed about the research. I think the other part, too, is the facilitator themselves being able to create a connection with people. And, the third thing is for people to feel like what they're saying is going to be heard, and also they will be informed about the outcomes or the results of the questionnaires or the focus groups". –[Male, age 66]<br><br>"I think one thing is educating people. Being transparent when doing research because a lot of the times people go into communities, they do research, and they take the valuable research. They don't give anything back, they don't tell them what the end results were. So being transparent, informing people, truly informing them from the beginning to the end of the process. Reaching out to the African-American community in different ways to let them know about the different projects that may be… different research that may be going on that they could be involved in. And informing them on how that could affect their health or their well-being by being a part of the solution to whatever problem it is." –[Female, age 34] |
| **Trust** | "Well, I think one of the most important things is trust. You know, having someone from some organization that they have trust in that's going to be very fair and attentive to the individual needs if they agreed to participate in the research. Just being… knowing that it's going to be used for good, for the right purpose." [Male, age 72] |
